# Supplementary material for: Crosstalk of growth factor receptors at plasma membrane clathrin-coated sites
Source: bioRxiv. 2024 May 18:2024.05.16.594559. Preprint. [Version 1] doi: 10.1101/2024.05.16.594559 (PMC11188102; doi:10.1101/2024.05.16.594559)
Supplement: Supplement 1 — SI Appendix Fig. 1. Quantitative measurements of EGF-induced changes to protein correlation with clathrin-coated sites. Automated correlation analysis values of 53 different fluorescently tagged proteins and individual clathrin sites in unstimulated (black) and EGF-stimulated (red) HSC3 cells with a 12 pixel-diameter analysis region. Error is Standard Deviation. SI Appendix Table 1. List of plasmids used in the imaging screen. [file NIHPP2024.05.16.594559v1-supplement-1.pdf]

bioRxiv preprint doi: https://doi.org/10.1101/2024.05.16.594559; this version posted May 18, 2024. The copyright holder for this preprint (which was not certified by peer review) is the author/funder. This article is a US Government work. It is not subject to copyright under 17 USC 105 and is also made available for use under a CC0 license.

SI Appendix Figure 1

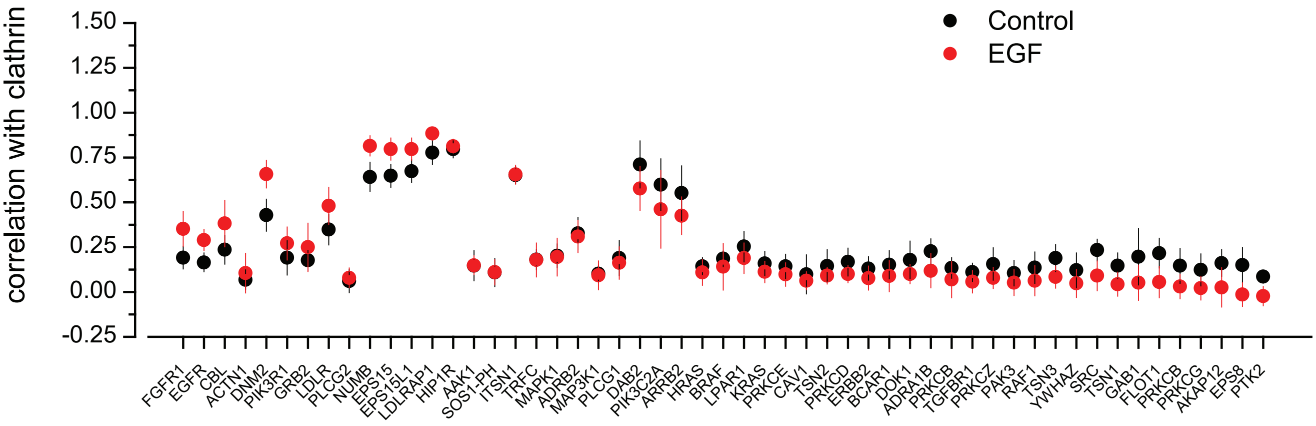

bioRxiv preprint doi: https://doi.org/10.1101/2024.05.16.594559; this version posted May 18, 2024. The copyright holder for this preprint (which was not certified by peer review) is the author/funder. This article is a US Government work. It is not subject to copyright under 17 USC 105 and is also made available for use under a CC0 license.

SI Appendix, Table 1

	HUGO (human gene symbol)	Plasmid name	Common full protein name	Tag	Source
1	ERBB2	Her2-GFP	Human epidermal growth factor receptor 2	GFP	Addgene 39321
2	PIK3R1	GFP-PI3Kp85	Phosphatidylinositol 3-kinase regulatory subunit p85	GFP	Taraska Lab*
3	PRKCB	PKCbII-mCh	Protein kinase c beta II	mCh	Taraska Lab
4	PRKCE	PKCe-mCh	Protein kinase c epsilon	mCh	Taraska Lab
5	PRKCG	PKCg-mCh	Protein kinase c gamma	mCh	Taraska Lab
6	PRKCD	PKCd-mCh	Protein kinase c delta C1 domain from PKCdelta (binds DAG)	mCh	Taraska Lab
7	PRKCZ	PKCz-mCh	Protein kinase c zeta	mCh	Taraska Lab
8	PTK2	FAK-mCh	Focal adhesion kinase 1	mCh	Addgene 55044
9	DAB2	Dab2-mCh	Disabled homolog adaptor protein 2	mCh	Taraska Lab
10	PLCG2	PLCg2-mCh	1-phosphatidylinositol 4,5-bisphosphate phosphodiesterase gamma-2	mCh	Taraska Lab
11	CBL	Cbl-mCh	E3 ubiquitin-protein ligase CBL	mCh	Taraska Lab
12	EPS8	Eps8-mCh	Epidermal growth factor receptor kinase substrate 8	mCh	Addgene 29779
13	ITSN1	Intersectin1-mCh	Intersectin 1	mCh	Taraska Lab
14	EPS15L1	Eps15R-mCh	Epidermal growth factor receptor substrate 15-like 1	mCh	Taraska Lab
15	ADRA1B	a1B-AR-GFP	alpha1-B adrenergic receptor	GFP	J Adolfo García-Sáinz (UNAM)
16	LPAR1	LPAR1-GFP	Lisophosphatidic acid receptor 1	GFP	J Adolfo García-Sáinz (UNAM)
17	HRAS	H-Ras-GFP	GTPase HRas	GFP	Addgene 18662
18	BCAR1	p130CAS-GFP	Breast cancer anti-estrogen resistance protein 1	GFP	Taraska Lab*
19	PAK4	PAK4-GFP	Serine/threonine-protein kinase PAK 4	GFP	Taraska Lab*
20	PLCG1	PLCg1-GFP	1-phosphatidylinositol 4,5-bisphosphate phosphodiesterase gamma-1	GFP	Taraska Lab*
21	AAK1	AAK1-GFP	AP2 Associated Kinase 1	GFP	Taraska Lab*
22	FGFR1	FGFR1-GFP	Fibroblast growth factor receptor 1	GFP	Taraska Lab*
23	RAF1	c-Raf1-GFP	RAF proto-oncogene serine/threonine-protein kinase	GFP	Taraska Lab*
24	GRB2	Grb2-GFP	Growth factor receptor-bound protein 2	GFP	Taraska Lab*

bioRxiv preprint doi: https://doi.org/10.1101/2024.05.16.594559; this version posted May 18, 2024. The copyright holder for this preprint (which was not certified by peer review) is the author/funder. This article is a US Government work. It is not subject to copyright under 17 USC 105 and is also made available for use under a CC0 license.

25	PRKCB	PKCbI-GFP	Protein kinase c beta I	GFP	Addgene 112265
26	DOK1	Dok1-GFP	Docking protein 1	GFP	Addgene 174194
27	EGFR	EGFR-GFP	Epidermal growth factor receptor 1	GFP	Addgene 32751
28	PIK3C2A	GFP-PIK3C2A	Phosphatidylinositol 4-phosphate 3-kinase C2 domain-containing subunit alpha	GFP	Addgene 161988
29	TNS3	Tensin3-GFP	Tensin 3	GFP	Addgene 105299
30	TNS2	Tensin2-GFP	Tensin 2	GFP	Addgene 105298
31	TFRC	TfR-GFP	Transferrin receptor protein 1	GFP	Taraska Lab
32	LDLRAP1	ARH-turboGFP	Low density lipoprotein receptor adapter protein 1	tGFP	Origene RG206643
33	ADRB2	Beta2-AR-GFP	Beta-2 adrenergic receptor	GFP	Taraska Lab
34	MAPK1	ERK2-GFP	Mitogen-activated protein kinase 1	GFP	Addgene 37145
35	FLOT1	Flotillin-GFP	Flotillin-1	GFP	Taraska Lab
36	TNS1	Tensin1-GFP	Tensin 1	GFP	Addgene 105297
37	EPS15	Eps15-GFP	Epidermal growth factor receptor substrate 15	GFP	Taraska Lab*
38	LDLR	LDLR-GFP	Low-density lipoprotein receptor	GFP	Thomas G. Jensen (Aarhus University)
39	YWHAE	GFP-14-3-3z	14-3-3 protein epsilon	GFP	Taraska Lab*
40	BRAF	GFP-BRAF	Serine/threonine-protein kinase B-raf	GFP	Taraska Lab*
41	AKAP12	gravin-GFP	A-kinase anchor protein 12	GFP	J. Scott Lab (UW)
42	KRAS	GFP-KRAS	GTPase KRas	GFP	Taraska Lab*
43	SOS1	SOS1-PH-mCh	Son of sevenless homolog 1 PH domain	mCh	Taraska Lab*
44	TGFBR1	TGFbR1-GFP	Transforming growth factor beta receptor 1	mCh	Addgene 54969
45	ACTN1	a-actinin-GFP	Alpha-actinin-1	GFP	Addgene 11908
46	ARRB2	b-arr2-GFP	Beta-arrestin-2	GFP	addgene
47	DNM2	Dyn2-GFP	Dynamin-2	GFP	Taraska Lab
48	HIP1R	HIP1R-mCh	Huntingtin-interacting protein 1-related protein	mCh	Taraska Lab
49	SRC	Src-GFP	Proto-oncogene tyrosine-protein kinase Src	GFP	Addgene 110496
50	CAV1	Cav1-GFP	Caveolin 1	GFP	Addgene 27704

bioRxiv preprint doi: https://doi.org/10.1101/2024.05.16.594559; this version posted May 18, 2024. The copyright holder for this preprint (which was not certified by peer review) is the author/funder. This article is a US Government work. It is not subject to copyright under 17 USC 105 and is also made available for use under a CC0 license.

51	GAB1	Gab1-GFP	GRB2 Associated Binding Protein 1	GFP	LSBio LS-N55372-1
52	NUMB	Numb-GFP	Protein numb homolog	GFP	Taraska Lab*
53	CLTA	mSca-CLC	Clathin light chain a	mSca	Taraska Lab
54	CLTA	GFP-CLC	Clathin light chain a	GFP	Taraska Lab
55	MAP3K1	MEK1-GFP	Mitogen-activated protein kinase kinase kinase 1	GFP	Addgene 14746
56	EGFR	EGFR-GFP	Epidermal growth factor receptor	GFP	Addgene 32751
57	EGFR	EGFR-mSca	Epidermal growth factor receptor	mSca	Taraska Lab

GFP: green fluorescent protein

tGFP: turbo green fluorescent protein

mCh: mCherry fluorescent protein

mSca: mScarlet fluorescent protein

*Asterisk indicates plasmid engineered for this paper
